# TGF-β1 Augments the Apical Membrane Abundance of Lemur Tyrosine Kinase 2 to Inhibit CFTR-Mediated Chloride Transport in Human Bronchial Epithelia

**DOI:** 10.3389/fcell.2020.00058

**Published:** 2020-02-07

**Authors:** Daniel F. Cruz, Nilay Mitash, Carlos M. Farinha, Agnieszka Swiatecka-Urban

**Affiliations:** ^1^BioSystems and Integrative Sciences Institute, Faculty of Sciences, University of Lisbon, Lisbon, Portugal; ^2^Department of Pediatrics, UPMC Children’s Hospital of Pittsburgh, University of Pittsburgh School of Medicine, Pittsburgh, PA, United States

**Keywords:** LMTK2, TGF-β1, bronchial epithelial cells, endocytosis, recycling, cystic fibrosis

## Abstract

The most common disease-causing mutation in the *Cystic Fibrosis Transmembrane Conductance Regulator (CFTR)* gene, F508del, leads to cystic fibrosis (CF), by arresting CFTR processing and trafficking to the plasma membrane. The FDA-approved modulators partially restore CFTR function and slow down the progression of CF lung disease by increasing processing and delivery to the plasma membrane and improving activity of F508del-CFTR Cl^–^ channels. However, the modulators do not correct compromised membrane stability of rescued F508del-CFTR. Transforming growth factor (TGF)-β1 is a well-established gene modifier of CF associated with worse lung disease in F508del-homozygous patients, by inhibiting CFTR biogenesis and blocking the functional rescue of F508del-CFTR. Lemur tyrosine kinase 2 (LMTK2) is a transmembrane protein localized at the apical and basolateral membrane domain of human bronchial epithelial cells. Phosphorylation of the apical membrane CFTR by LMTK2 triggers its endocytosis and reduces the abundance of membrane-associated CFTR, impairing the CFTR-mediated Cl^–^ transport. We have previously shown that LMTK2 knockdown improves the pharmacologically rescued F508del-CFTR abundance and function. Thus, reducing the LMTK2 recruitment to the plasma membrane may provide a useful strategy to potentiate the pharmacological rescue of F508del-CFTR. Here, we elucidate the mechanism of LMTK2 recruitment to the apical plasma membrane in polarized CFBE41o- cells. TGF-β1 increased LMTK2 abundance selectively at the apical membrane by accelerating its recycling in Rab11-positive vesicles without affecting LMTK2 mRNA levels, protein biosynthesis, or endocytosis. Our data suggest that controlling TGF-β1 signaling may attenuate recruitment of LMTK2 to the apical membrane thereby improving stability of pharmacologically rescued F508del-CFTR.

## Introduction

Cystic fibrosis (CF), the most common autosomal recessive disease in Caucasians, is caused by mutations in the *cystic fibrosis transmembrane conductance regulator (CFTR)* gene that encodes a cyclic adenosine monophosphate (cAMP)-activated anion channel. CFTR is expressed at the apical plasma membrane of epithelial cells in most tissues, including the airway (Andersen, 1938; Boucher et al., 1983; Riordan et al., 1989; Collins, 1992). In human bronchial epithelial (HBE) cells, CFTR regulates mucociliary clearance by maintaining the airway surface liquid (ASL) homeostasis (Regnis et al., 1994; Boucher, 2004). The most common disease-causing mutation present on at least one allele in 90% of CF patients is the deletion of Phe508 (F508del), caused by an in-frame deletion of three nucleotides (Feriotto et al., 1999). This mutation causes a biosynthetic processing defect leading to intracellular retention of CFTR protein and severely impairs the CFTR channel function (Penque et al., 2000). The Food and Drug Administration (FDA)-approved correctors rescue the biosynthetic processing of F508del-CFTR protein while potentiators improve the rescued channel function (Molinski et al., 2012). VX-809 (Lumacaftor) and VX-661 (Tezacaftor) are FDA-approved CFTR correctors that when combined with the potentiator VX-770 (Ivacaftor) modestly reduced exacerbation rates and respiratory symptoms (Donaldson et al., 2013; Wainwright et al., 2015; Ratjen et al., 2017). The new-generation correctors, VX-659 and VX-445 have recently demonstrated profound clinical promise because of additive benefit when combined with the dual therapy with VX-661/770 (Davies et al., 2018; Keating et al., 2018). The *transforming growth factor (TGF)-*β*1* gene is a known modifier associated with worse lung disease in CF patients homozygous for F508del (Drumm et al., 2005; Bremer et al., 2008; Cutting, 2010). Published data show that TGF-β1 reduces CFTR mRNA levels and prevents the corrector/potentiator mediated rescue of the CFTR channel function in primary differentiated HBE cells homozygous for the F508del (Roux et al., 2010; Snodgrass et al., 2013; Sun et al., 2014). Thus, TGF-β1 may compromise the full beneficial effect of the corrector/potentiator therapy in the CF patients who have increased TGF-β1 signaling due to the *TGF-*β*1* gene polymorphisms, lung infection or environmental factors (Arkwright et al., 2000; Drumm et al., 2005; Collaco et al., 2008; Cutting, 2015).

In addition to the role in CF, TGF-β1 is a critical mediator in chronic obstructive pulmonary disease (COPD), contributing to acquired CFTR dysfunction (Takizawa et al., 2001; Mak et al., 2009; Morty et al., 2009; Dransfield et al., 2013; Sailland et al., 2017). TGF-β1 also plays central role in the early phase of acute lung injury, leading to development of pulmonary edema by two mechanisms (Hurst et al., 1999; Pittet et al., 2001; Hamacher et al., 2002; Fahy et al., 2003). First, TGF-β1 decreases the airspace fluid clearance by reducing the apical abundance of epithelial sodium channel (ENaC) via extracellular signal-regulated kinase (ERK)1/2 dependent mechanism (Frank et al., 2003). Second, TGF-β1 inhibits the β-adrenergic agonist-stimulated CFTR-dependent alveolar fluid clearance via phosphatidylinositol 3-kinase (PI3K)-dependent inhibition of CFTR protein biosynthesis and channel function (Roux et al., 2010).

Cystic fibrosis transmembrane conductance regulator interactor lemur tyrosine kinase 2 (LMTK2), despite its name, is a transmembrane serine/threonine kinase involved in intracellular signaling, protein trafficking, apoptosis, and cell differentiation (Wang and Brautigan, 2002; Kesavapany et al., 2003; Kawa et al., 2004; Inoue et al., 2008). We have shown that LMTK2 mediates an inhibitory phosphorylation of membrane-resident CFTR-Ser^737^, leading to its endocytosis and inhibition of CFTR-mediated Cl^–^ transport (Luz et al., 2014). The pre-clinical relevance of the finding is that LMTK2 depletion increased the efficacy of Lumacaftor in HBE cells from F508del homozygous lungs (Luz et al., 2014). Although little is known about the interface between LMTK2 and TGF-β1, it has been shown that the kinase facilitates Smad2 signaling (Manser et al., 2012).

In view of the role of TGF-β1 in CFTR dysfunction in different forms of lung disease, our aim was to determine whether TGF-β1 may inhibit the function of membrane-resident CFTR channels via LMTK2 dependent mechanism in HBE cell models. Here, we report that TGF-β1 augments the apical membrane abundance of LMTK2 by increasing its recycling in Rab11-dependent manner. The physiologic consequence of the TGF-β1 effect is inhibition of CFTR mediated Cl^–^ transport. We propose that one of the mechanisms through which TGF-β1 inhibits CFTR mediated Cl^–^ transport is by increasing the apical membrane density of LMTK2 that, in turn, phosphorylates membrane-resident CFTR-Ser^737^ inducing its endocytosis.

## Materials and Methods

### Cell Lines and Cell Culture

Primary differentiated HBE cells were received from the CF Research Center Epithelial Cell Core at the University of Pittsburgh, School of Medicine, Pittsburgh, PA, United States as previously described (Myerburg et al., 2010; Snodgrass et al., 2013; Luz et al., 2014). The Epithelial Cell Core procures these cells from excess pathologic lung tissue from explanted human lungs following lung transplantation at the University of Pittsburgh Medical Center under an approved IRB protocol. The F508del HBE cells were derived from lungs homozygous for the F508del mutation. Cells were prepared using previously described methods approved by the University of Pittsburgh Institutional Review Board (Myerburg et al., 2010). Subsequently, cells were cultured on collagen-coated Transwell filters (Corning, Corning, NY, United States) (0.33 cm^2^ at density of ∼2 × 10^5^/cm^2^) and maintained in air-liquid interface (ALI) for 6 weeks while differentiation media was changed basolaterally twice weekly. Parental human bronchial epithelial CFBE41o- cells were seeded on collagen-coated Transwell filters and cultured in ALI for 6–9 days (Ye et al., 2010; Cihil et al., 2012; Luz et al., 2014). In some experiments, denoted below, CFBE41o- cells were cultured on tissue culture plates. Fetal bovine serum (FBS) was removed from the media 24 h before experiments to avoid any residual TGF-β1, increase cell polarization, and to promote cell cycle synchronization.

### Antibodies and Reagents

The antibodies used were polyclonal anti-LMTK2 (SAB4500900, Sigma-Aldrich, St. Louis, MO, United States) and monoclonal anti-Na+/K+ ATPase a-1 (clone C464.6, Merck KGaA, Darmstadt, Germany), monoclonal anti-Rab5 (Cat. 610282) and monoclonal anti-Ezrin (Cat. 610603, BD Biosciences, San Jose, CA, United States), polyclonal anti-Rab11A (Cat. 71-5300, Thermo Fisher Scientific, Waltham, MA, United States), and goat anti-mouse and goat anti-rabbit horseradish peroxidase-conjugated secondary antibodies (Bio-Rad Laboratories, Hercules, CA, United States). All antibodies were used at the concentrations recommended by the manufacturer or as indicated in the figure captions. Human TGF-β1 (Sigma-Aldrich) was resuspended in the vehicle containing 4 mM HCl and 1 mg/mL bovine serum albumin (BSA, Sigma-Aldrich) and used at a concentration 15 ng/ml.

### Real-Time Quantitative Reverse-Transcription PCR (qRT-PCR)

Real time reactions were run in triplicates with each reaction emanating from a starting sample amount of 20 ng total RNA before Reverse Transcription to cDNA. Superscript II Reverse Transcriptase (Thermo Fisher Scientific) was used to generate cDNA from total RNA. qRT-PCR was performed using ABsolute^TM^ Blue QPCR SYBR^®^ Green ROX Mix (Thermo Fisher Scientific) and ABI PRISM^®^ 7300 Sequence Detection System (Applied Biosystems, Foster City, CA, United States) according to the manufacturer’s instructions. The primer sequences for LMTK2 were from the Harvard Medical School Primer Bank (LMTK2-688F forward: 5′-TTGCCCGCCACAGTCTAAAC-3′ and LMTK2-770R reverse: 5′-GATGACTCTTGCTACGCTAGT-3′). Fluorescence emission was detected for each PCR cycle, and the threshold cycle (Ct) values and the average Ct of the triplicate reactions were determined for CFTR and the reference gene GAPDH. The Ct value was defined as the actual PCR cycle when the fluorescence signal increased above the threshold, and the ΔCt was determined for each sample by subtracting the Ct for GAPDH from the Ct for CFTR, and the average ΔCt of the triplicate samples was determined. The ΔΔCt was calculated by subtracting the ΔCt for the vehicle treated cells from the ΔCt for the TGF-β1 treated cells. Fold change values were determined according to the following formula: fold change = 2^–ΔΔ*Ct*^. Log2FC (Log2 fold change) was calculated by converting fold change value in log base 2.

### Biochemical Determination of the Plasma Membrane Protein Abundance and Western Blotting

Detection of plasma membrane LMTK2 was performed by domain selective plasma membrane biotinylation, as previously described (Swiatecka-Urban et al., 2002). CFBE41o- cells were treated with TGF-β1 or vehicle for 1 h. Apical or basolateral plasma membrane proteins of ALI cultures of HBE or CFBE41o- cells were selectively biotinylated using cell membrane impermeable EZ-Link^TM^ Sulfo-NHS-LC-Biotin (Pierce Chemical Co., Dallas, TX, United States), followed by cell lysis in buffer containing 25 mM HEPES, 10% v/v glycerol, 1% v/v Triton X-100, and Complete Protease Inhibitor Mixture (Roche Applied Sciences, Indianapolis, IN, United States). Lysates were centrifuged at 14,000 x *g* and biotinylated proteins were isolated from the supernatants, considered as whole cell lysates (WCL), by incubation with streptavidin-agarose beads, eluted into 2x Laemmli sample buffer (Bio-Rad Laboratories) containing DL-dithiothreitol (DTT; Sigma-Aldrich), and separated by 7.5% sodium dodecyl sulfate polyacrylamide gel electrophoresis (SDS-PAGE; Bio-Rad Laboratories). The immunoreactive bands were visualized by western blotting (WB) with Western Lightning Chemiluminescence Reagent Plus (PerkinElmer LAS Inc., Boston, MA, United States). Protein abundance was quantified by densitometry using exposures within the linear dynamic range of the film.

### Immunohistochemistry (IHC)

Formalin-fixed, paraffin-embedded tissue sections were stained on the Ventana BenchMark Ultra automated staining platform (Ventana Medical Systems Inc., Tucson, AZ, United States) as recently described (Mitash et al., 2019). Slides were pretreated with ULTRA cell conditioning solution CC1 (Ventana) for 64 min and stained using mouse monoclonal primary antibody against LMTK2 (Acris AM20991PU-N), at 1:100 dilution. OptiView 3,3′-diaminobenzidine (DAB) IHC detection kit with OptiView amplification indirect, biotin-free multimer amplification system (Ventana) was used for detection of primary antibodies. All slides were counterstained with hematoxylin and routinely dehydrated, cleared, and coverslipped in resinous mounting media.

### Endocytic Assay

Endocytic assays were performed in CFBE41o- cells, as previously described with some modifications (Cihil et al., 2012; Cihil and Swiatecka-Urban, 2013). The apical or basolateral plasma membrane proteins were selectively biotinylated at 4°C using cleavable EZ-Link^TM^ Sulfo-NHS-SS-Biotin (Pierce Chemical Co.) and excess biotin was removed after biotinylation by washing with cold phosphate-buffered saline (PBS; Thermo Fisher Scientific), containing Ca^+2^ and Mg^+2^ (PBS^++^). To enable endocytic trafficking, cells were quickly moved to a 37°C bath of PBS^++^ containing in basolateral side TGF-β1 or vehicle for 5, 10 or 15 min. Subsequently, disulfide bonds on Sulfo-NHS-SS-biotinylated proteins remaining at the plasma membrane were reduced by L-glutathione (GSH; Sigma-Aldrich) at 4°C. At this point, biotinylated proteins reside within the endocytic compartment. Cells were lysed and biotinylated proteins were isolated by streptavidin-agarose beads. The amount of biotinylated LMTK2 at 4°C and without the 37°C warming was considered 100%. The amount of biotinylated LMTK2 remaining at the plasma membrane after GSH treatment at 4°C and without the treatment at 37°C was considered background and was subtracted from the biotinylated LMTK2 after warming to 37°C at each time point.

### Cell Fractionation to Isolate the Cytosolic and Membrane Fraction

CFBE41o- cells were washed with PBS^++^, scrapped and centrifuged at 200 x *g*. After centrifugation, cells were resuspended in homogenization buffer (HB) containing 50 mM MOPS-NaOH, 125 mM NaCl, 1 mM EGTA, 0.1% 2-mercaptoethanol and Complete protease inhibitor mixture. Homogenates were centrifuged at 100,000 x *g* at 4°C for 20 min, using a Sorvall WX80 Ultra centrifuge. Supernatant containing the cytosolic fraction was collected. Pellet was resuspended in HB buffer, applied on top of 28% sucrose layered on 50% sucrose and centrifuged at 100,000 x *g* at 4°C for 40 min. The 28/50% interface was collected and washed by centrifugation with HB buffer. Pellet was resuspended with HB buffer with 1% Igepal (Sigma-Aldrich) and centrifuged at 16,000 x *g* for 15 min at 4°C. The resulting supernatant containing cellular membrane fraction was collected. The cytosolic and membrane fractions were mixed with 2x Laemmli sample buffer containing DTT and analyzed by WB.

### RNA-Mediated Interference

Transfection of CFBE41o- cells with siRNA targeting human *Rab5* gene (siRab5, siGENOME Human Rab5 siRNA; Dharmacon, Cambridge, United Kingdom), human *Rab11A* gene (siRab11 Accell Human Rab11A siRNA; Dharmacon) or non-targeting siRNA (siCTRL, siGENOME Non-Targeting Pool #2; Dharmacon) was performed using Lipofectamine RNAiMAX Transfection Reagent (Thermo Fisher Scientific), according to the manufacturer’s instructions. CFBE41o- cells were plated on collagen-coated cell culture plates and incubated with the optimized transfection mixture containing 50 nM of siRNA, at 37°C for 24 h. Next day, cells were transferred to collagen-coated Transwell filters for 5 days to allow cell polarization. Experiments were conducted 6 days after siRNA transfection.

### Short Circuit Recordings

The short circuit current (I_SC_) was measured under asymmetrical Cl^–^ conditions as previously described (Snodgrass et al., 2013). In brief, 6-week ALI cultures of HBE cells were mounted in Ussing-type chambers (Physiological Instruments, San Diego, CA, United States). Transepithelial resistance was measured by periodically applying a 1-mV voltage pulse and was calculated using Ohm’s law. The composition of the bathing Ringer’s solutions were as follows, apical: 115 mM NaCl, 25 mM NaHCO_3_, 5.0 mM KCl, 10 mM HEPES, 1.0 mM MgCl_2_, 1.5 mM CaCl_2_, and 5.0 mM glucose; and basolateral 114 mM Na gluconate, 25 mM NaHCO_3_, 5.0 mM KCl, 10 mM HEPES, 1.0 mM MgCl_2_, 1.5 mM CaCl_2_, and 5.0 mM glucose. Chambers were constantly gassed with a mixture of 95% O_2_ and 5% CO_2_ at 37°C, which maintained the pH at 7.4. Following a 5-min equilibration period, the baseline I_SC_ was recorded. Amiloride (50 μM) was added to the apical bath solution to inhibit Na^+^ absorption through ENaC. Subsequently, CFTR mediated Isc was stimulated with the cAMP agonist forskolin (20 μM) together with the IBMX (1 mM) to prevent cAMP hydrolysis, added to the apical and basolateral bath solutions, and subsequently inhibited by thiazolidinone CFTR_inh_-172 (5 μM) added to the apical bath solution. Data are expressed as the forskolin/IBMX stimulated Isc, calculated by subtracting the peak-stimulated Isc from the baseline Isc after amiloride treatment.

### Data Analysis and Statistics

Statistical analysis of the data was performed using GraphPad Prism version 8.0 for Windows (GraphPad Software Inc., San Diego, CA, United States). Data are expressed as mean ± standard error of the mean (SEM). The means were compared by a two-tailed *t*-test. *p* value < 0.05 was considered significant where ^∗^*p* < 0.05; ^∗∗^*p* < 0.01.

## Results

### TGF-β1 Selectively Increases LMTK2 Abundance at the Apical Plasma Membrane by Post-transcriptional Mechanisms

**FIGURE 1:**
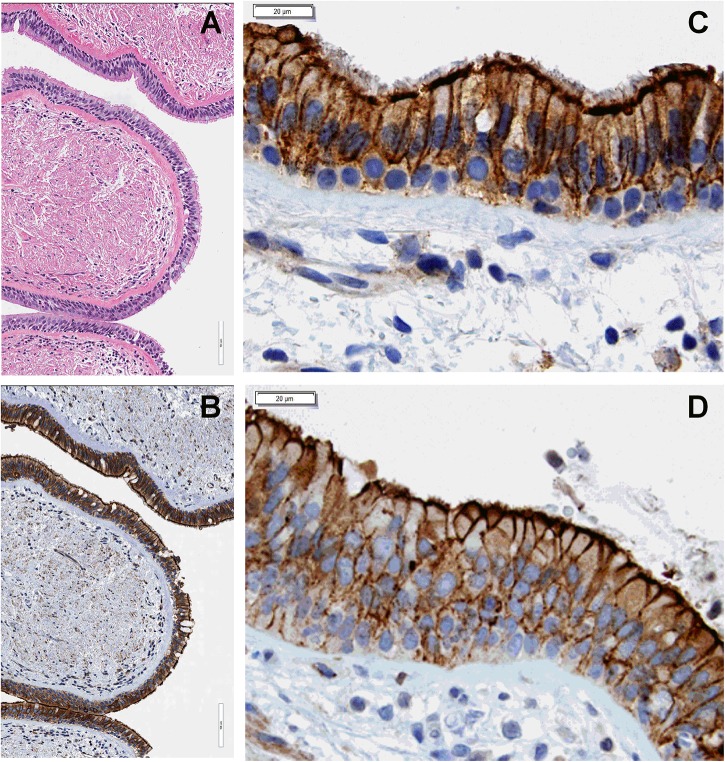
IHC experiments demonstrating subcellular localization of endogenous LMTK2 in human bronchial tissue. LMTK2 was detected with antibody AM20991PU-N. Hematoxylin stain **(A)** showing the ultrastructure of human bronchial tissue (20x) from control lung and the LMTK2 staining in the tissue at 20x **(B)**. Images (40x), showing that LMTK2 assumes granular intracellular distribution and is enriched in the apical and basolateral membrane in control **(C)**, and F508del bronchial epithelium **(D)**. Experiments were performed in triplicates in tissues from five lungs per condition with similar results.

We have previously shown that LMTK2 is present at the apical and basolateral membrane domain in bronchial epithelial cell models used in our studies, HBE and CFBE41o- cells, and triggers CFTR endocytosis from the apical membrane by phosphorylating CFTR-Ser^737^ (Luz et al., 2014). We first validated the cell models for subsequent LMTK2 studies and examined the subcellular localization of endogenous LMTK2 in human bronchial tissue. Evaluation by IHC demonstrated that LMTK2 assumes granular intracellular distribution and is enriched in the apical and basolateral membrane, consistent with the localization determined by selective cell surface biotinylation in HBE and CFBE41o- cells (Figure 1). There was no obvious difference in the subcellular localization of LMTK2 between tissues from lungs homozygous for F508del and controls without disease.

**FIGURE 2:**
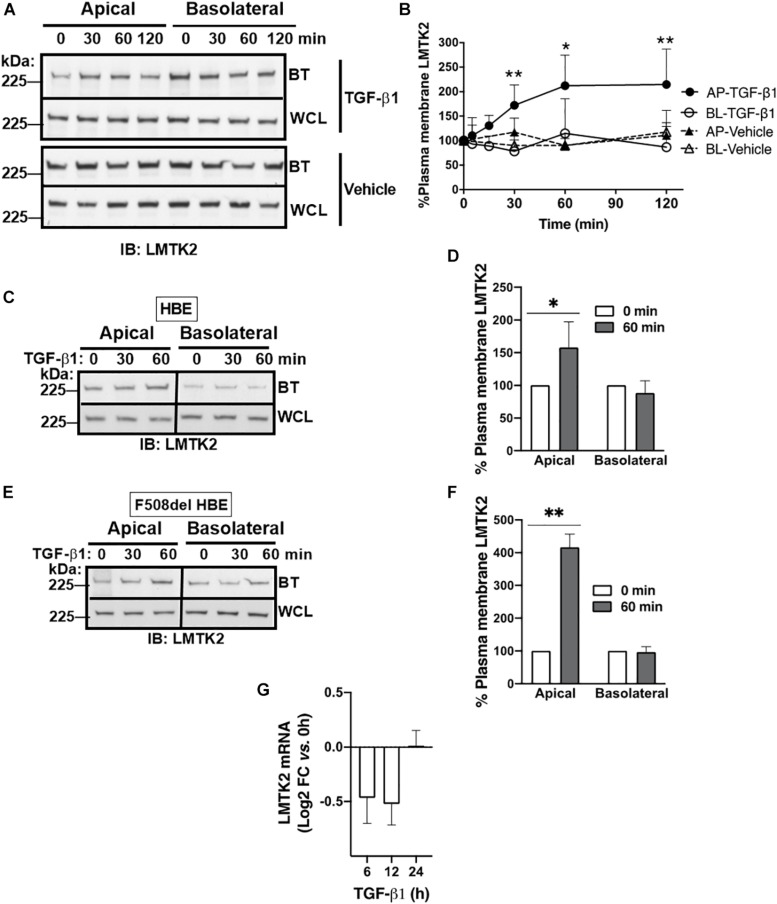
TGF-β1 increased LMTK2 abundance at the apical plasma membrane of polarized CFBE41o- and differentiated HBE cells. Representative immunoblots **(A)** and summary of experiments **(B)** demonstrating increased LMTK2 levels at the apical membrane after 30 min of TGF-β1 stimulation, and reaching maximum at 1 h. CFBE41o- cells were cultured on Transwell filters to allow polarization and treated with TGF-β1 (15 ng/ml) or vehicle control for 5, 15, 30, 60 or 120 min. Plasma membrane proteins were isolated by cell surface biotinylation and LMTK2 abundance in each domain was assessed by WB. LMTK2 expression in whole cell lysates (WCL) was used as loading control. 3–7 experiments/group. Additionally, TGF-β1 treatment for 60 min increased the apical membrane LMTK2 in HBE **(C,D)** and F508del HBE cells **(F,G)** without affecting the basolateral membrane LMTK2. LMTK2 expression in WCL was used as a loading control. Five replicates from three HBE cell donors **(C,D)** and three replicates from one F508del HBE cell donor **(E,F)**. qRT-PCR experiments demonstrating that TGF-β1 did not increase the LMTK2 mRNA level in HBE cells **(G)**. TGF-β1 (15 ng/ml) was added to the basolateral medium and cells were incubated for 6, 12 or 24 h. Raw data were analyzed using the ΔΔCt method. Changes in the LMTK2 mRNA were normalized to GAPDH and expressed as Log2 FC versus untreated cells (time zero). Experiments were performed in triplicates in HBE cells from 4 lung donors. ^∗^*p* < 0.05 and ^∗∗^*p* < 0.01 versus vehicle. Error bars, SEM. AP, apical; BL, basolateral; BT, biotinylation; IB, immunoblot; WCL, whole cell lysate.

Next, we examined whether TGF-β1 affects the plasma membrane abundance of LMTK2. ALI cultures of CFBE41o- cells were treated with TGF-β1 (15 ng/ml) or vehicle added to the basolateral cell culture medium for up to 120 min and the plasma membrane abundance of LMTK2 was examined by domain-selective cell surface biotinylation, as previously described (Cihil and Swiatecka-Urban, 2013; Luz et al., 2014). We observed rapid and selective increase of LMTK2 levels in the apical membrane, reaching significance at 30 min and maximum at 60 min after TGF-β1 treatment (Figures 2A,B). TGF-β1 did not increase the total cellular abundance of LMTK2 by 120 min demonstrating that the apical membrane increase did not result from inhibiting the LMTK2 protein degradation (Figure 2A). As TGF-β1 did not change the basolateral membrane LMTK2 abundance, this suggests that the apical membrane increase of LMTK2 is domain-selective and not mediated by the basolateral-to-apical transcytosis of LMTK2. Next, we validated the results from an immortalized cell model in the primary differentiated HBE cells. Cells originated from the non-CF (HBE) or CF lungs (CF HBE) cultured in ALI for 6 weeks to full differentiation were treated with TGF-β1 or vehicle for 60 min as described above. TGF-β1 selectively increased the apical membrane abundance of LMTK2 in HBE and CF HBE cells (Figures 2C–F). To examine TGF-β1 effects on LMTK2 mRNA level, HBE cells were treated with TGF-β1 or vehicle for up to 24 h, total RNA was isolated and LMTK2 mRNA was quantified by qRT-PCR. TGF-β1 did not increase LMTK2 mRNA level (Figure 2G). Taken together, the rapid augmentation of LMTK2 abundance in the apical membrane and lack of effect at the LMTK2 mRNA level strongly suggest that TGF-β1 induces post-translational modifications or endocytic trafficking of LMTK2 to increase its apical membrane abundance.

### TGF-β1 Inhibits CFTR Mediated Isc, Temporally Correlating It With Increased Apical Membrane LMTK2 Abundance

**FIGURE 3:**
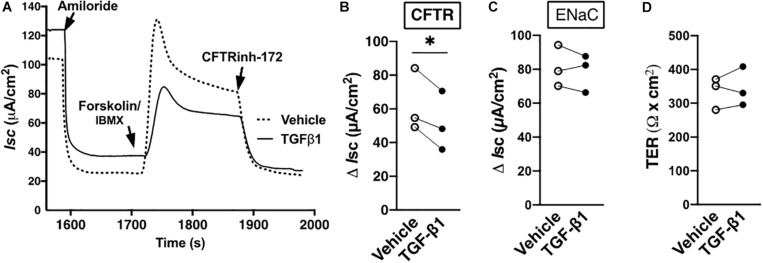
TGF-β1 inhibited CFTR mediated Isc in HBE cells within 60 min. Representative Ussing chamber recordings **(A)** and summary of data **(B)** showing that TGF-β1 decreased the CFTR mediated Isc in HBE cells, compared to vehicle control. TGF-β1 or vehicle control were added to the basolateral medium for 60 min. In the Ussing chamber, amiloride (50 μM) was added to the apical bath solution to inhibit ENaC. Forskolin (20 μM) and IBMX (1 mM) were added to increase cAMP level and activate CFTR, and CFTR_inh_-172 (5 μM) was added to inhibit CFTR channel function. The CFTR mediated Isc was calculated by subtracting the Isc after amiloride treatment from the peak forskolin/IBMX-stimulated Isc and expressed as ΔIsc. The 60-min TGF-β1 treatment did not change the ENaC mediated Isc calculated by subtracting the Isc after amiloride treatment from the baseline Isc before any treatment **(C)** or the transepithelial resistance (TER; **d**). Three monolayers per donor HBE cell line from three lung donors per group were used. Each gray circle in panels **(B–D)** represents the mean value from three monolayers per donor HBE cells. The means were compared by paired two-tailed *t*-test. Error bars, SEM. ^∗^*p* < 0.05.

It is expected that TGF-β1, by way of augmenting LMTK2 abundance in the apical plasma membrane, should also inhibit CFTR mediated Isc. Except for alveolar epithelial cells, little is known about the short-term effect of TGF-β1 on the CFTR mediated Cl^–^ transport (Roux et al., 2010). TGF-β1 or vehicle were added to the basolateral medium of HBE cells for 60 min, the time required for the maximal plasma membrane recruitment of LMTK2. The ENaC function was inhibited by amiloride and the CFTR channel was activated by forskolin in the presence of IBMX and it was inhibited with CFTR_Inh_-172. TGF-β1 inhibited the CFTR mediated Isc without affecting the ENaC mediated Isc in HBE cells from three different lung donors (Figure 3). These data temporally correlate the TGF-β1 augmentation of LMTK2 at the apical membrane with inhibition of CFTR mediated Cl^–^ transport.

### TGF-β1 Increases the Apical Membrane LMTK2 Abundance by Specifically Accelerating Its Recycling in Rab11-Positive Vesicles

**FIGURE 4:**
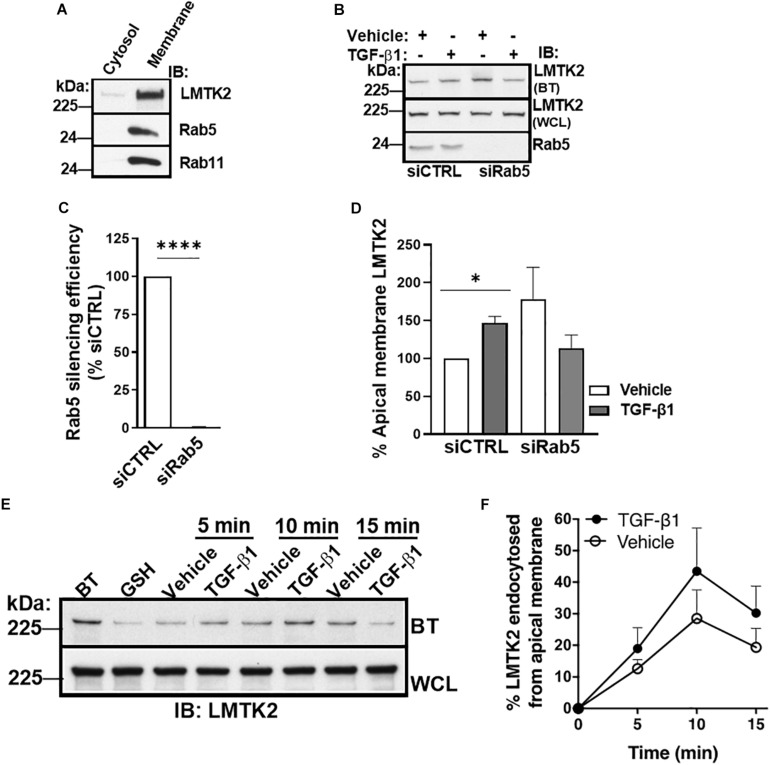
TGF-β1 did not inhibit LMTK2 endocytosis. Representative immunoblots of LMTK2, Rab11 and Rab5 abundance in both cytosol and membrane fractions **(A)**. CFBE41o- cells were cultured on Transwell filters to allow polarization. Subcellular fractionation was performed through ultracentrifugation (100,000 x *g*) and using discontinuous sucrose gradient (28%/50% sucrose interface). Cytosol and membrane fractions of the cells were isolated and protein levels were measured. LMTK2, Rab5 and Rab11 were enriched at the membrane fraction of the cells. Representative immunoblots **(B)** and summary of experiments **(C,D)** demonstrating that Rab5-dependent endocytosis is not affected by TGF-β1 stimulation. CFBE41o- cells were cultured on Transwell filters to allow polarization, transfected with Rab5-specific siRNA (siRab5) or non-targeting control siRNA (siCTRL) and treated with TGF-β1 (15 ng/ml) or its vehicle for 1 h. Apical membrane proteins were isolated by cell surface biotinylation. We observed an increase of LMTK2 abundance at the apical membrane after TGF-β1 in siNeg-transfected CFBE41o- cells that is disrupted after impairment of Rab5-dependent endocytosis. Apical LMTK2 levels were normalized to total LMTK2 levels present in the whole cell lysate. Three experiments/group. Representative immunoblots **(E)** and summary of experiments **(F)** showing the effects of TGF-β1 on CFTR endocytosis. Apical membrane proteins of polarized CFBE41o- cells were biotinylated, and endocytosis was induced at 37°C with TGF-β1 or vehicle treatment for 5, 10, and 15 min. LMTK2 endocytosis peaked at 10 min. TGF-β1 tended to increase the endocytosis. Internalized LMTK2 was normalized for total LMTK2 abundance. Five experiments/group. ^∗^*p* < 0.05; ^∗∗∗∗^*p* < 0.0001. Error bars, SEM. BT, biotinylation; IB, immunoblot; WCL, whole cell lysate.

Next, we examined TGF-β1 effects on the endocytic trafficking of LMTK2. In transfected HeLa cells, LMTK2 was found to be enriched in intracellular membranes associated with endocytic and recycling vesicles, namely the transferrin-, EEA1-, Rab5-, and Rab11-positive vesicles (Chibalina et al., 2007). However, the association of endogenous LMTK2 with these vesicular compartments is unknown in HBE cells. To determine the subcellular localization of LMTK2, Rab5 and Rab11, polarized CFBE41o- cells were homogenized and subjected to sucrose density gradient ultracentrifugation to isolate the membrane and cytosolic fraction. Endogenous LMTK2, Rab5 and Rab11 were specifically enriched in the membrane fraction in the HBE cell model (Figure 4A).

Subsequently, TGF-β1 effects on the endocytic trafficking of LMTK2 were studied by the siRNA-mediated silencing approach and selective apical membrane biotinylation. CFBE41o- cells were transfected with siRab5 or siCTRL. Transfected cells were plated on culture dishes and 24 h later sub-cultured to collagen-coated permeable Transwell filters for 5 days, the time required for cell polarization. Subsequently, cells were treated with TGF-β1 or vehicle for 60 min, followed by biotinylation of the apical membrane proteins, cell lysis, pull-down of biotinylated proteins with streptavidin beads, elution, and WB. The Rab5 silencing efficiency was approximately 99% (Figures 4B,C). If TGF-β1 increases the apical membrane LMTK2 level by inhibiting its endocytosis in Rab5-positive vesicles, it is expected that Rab5 knockdown would increase the apical membrane abundance of LMTK2 in the vehicle and TGF-β1 treated cells. As shown in Figures 4B,D, TGF-β1 increased LMTK2 abundance in the apical membrane of cells transfected with siCTRL while the highly efficient siRab5 did not increase LMTK2 levels in the apical membrane in cells treated with either vehicle or TGF-β1. These data suggest that the recruitment of LMTK2 to the apical membrane is Rab5-independent.

To examine more directly how TGF-β1 affects LMTK2 endocytosis we performed the GSH protection assay in polarized CFBE41o- cells (Cihil et al., 2012; Cihil and Swiatecka-Urban, 2013; Luz et al., 2014). Apical plasma membrane proteins were biotinylated with NHS-SS-Biotin, and endocytosis of biotinylated proteins was induced by incubation with TGF-β1 or vehicle at 37°C. Subsequently, membrane trafficking was stopped by rapid cooling to 4°C and the disulfide bond in NHS-SS-Biotin attached to the proteins still remaining at the apical membrane was reduced with GSH so that only proteins that were endocytosed from the apical membrane were protected from GSH and remained biotinylated. After cell lysis, biotinylated proteins were isolated by streptavidin beads, eluted into sample buffer and biotinylated LMTK2 was detected by WB. TGF-β1 did not decrease LMTK2 endocytosis (Figures 4E,F). These data strongly support a model that the TGF-β1-induced augmentation of apical membrane LMTK2 is not mediated by inhibiting LMTK2 endocytosis.

**FIGURE 5:**
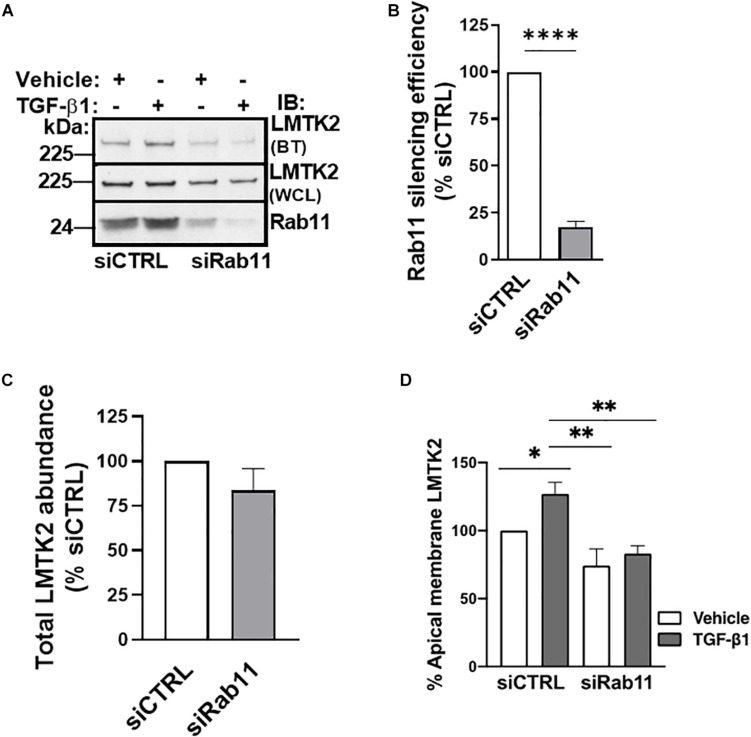
Rab11 knockdown blocked TGF-β1 recruitment of LMTK2 to the apical membrane. Representative immunoblots **(A)** and summary of experiments **(B–D)** demonstrating that TGF-β1 treatment increases apical LMTK2 abundance, in a process mediated by Rab11-dependent vesicular trafficking. CFBE41o- cells were cultured on Transwell filters to allow polarization, transfected with Rab11-specific siRNA (siRab11) or non-targeting control siRNA (siCTRL) and treated with TGF-β1 (15 ng/ml) or its vehicle control for 1 h. Apical membrane proteins were isolated by cell surface biotinylation. An increase of LMTK2 abundance at the apical membrane after TGF-β1 stimulation in siNeg-transfected CFBE41o- cells was observed; however, increased LMTK2 abundance is disrupted after impairment of Rab11-dependent recycling. Total levels of LMTK2 were not affected after siRab11 knockdown. Apical LMTK2 was normalized to total LMTK2 present in the whole cell lysate. Total LMTK2 abundance was normalized to ezrin in the whole cell lysate, used as a loading control. ^∗^*p* < 0.05; ^∗∗^*p* < 0.01, and ^∗∗∗∗^*p* < 0.0001. Three replicates. Error bars, SEM. BT, biotinylation; IB, immunoblot; WCL, whole cell lysate.

Next, we examined whether TGF-β1 increases the apical abundance of LMTK2 by stimulating its trafficking to the apical membrane in Rab11-positive vesicles. Rab11 is associated with the perinuclear recycling endosomes and regulates the recycling of endocytosed proteins to the apical plasma membrane (Ullrich et al., 1996; Takahashi et al., 2012). In addition, Rab11 controls vectorial transport from the *trans*-Golgi to the plasma membrane. Silencing of Rab11a was performed as described above for Rab5 with the efficiency exceeding 75% (Figures 5A,B). TGF-β1 increased LMTK2 abundance in the apical membrane in cells transfected with siCTRL while siRab11 eliminated the effect (Figures 5A,C). These results demonstrate that TGF-β1 is unable to increase the apical membrane LMTK2 after Rab11 knockdown. Taken together, the results demonstrate that TGF-β1 stimulates specifically the Rab11-dependent trafficking of LMTK2 to augment its apical membrane abundance, without inhibiting the LMTK2 endocytosis.

## Discussion

**FIGURE 6:**
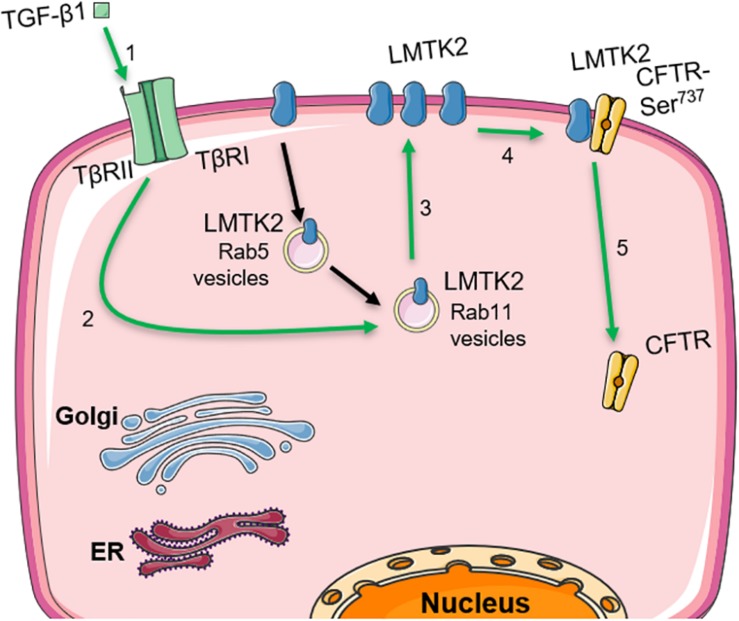
Model describing how TGF-β1 augments the apical membrane abundance of LMTK2 to inhibit CFTR mediated Cl^–^ transport in human bronchial epithelia. TGF-β1 stimulation (1) increases LMTK2 recycling through Rab11-positive vesicles (2), enhancing the recruitment of LMTK2 to the apical plasma membrane of HBE cells (3). At the apical plasma membrane, LMTK2 induces the inhibitory phosphorylation of CFTR-Ser^737^ (4), triggering CFTR endocytosis (5) and the inhibition of CFTR-mediated Cl^–^ transport. This figure was created with the aid of Servier medical art.

The work presented here connects the inhibitory effects of TGF-β1 and LMTK2 on CFTR function in HBE cells. Our data demonstrate that TGF-β1 acutely augments the apical membrane abundance of LMTK2 by increasing its recycling in Rab11-dependent manner and inhibits CFTR mediated Isc while our previously published work showed that LMTK2 promotes phosphorylation of the membrane-resident CFTR-Ser^737^ leading to CFTR endocytosis and decreased CFTR-mediated Cl^–^ transport (Figure 6) (Luz et al., 2014).

The above model is supported by data showing increased apical membrane LMTK2 abundance with concurrent inhibition of CFTR mediated Isc after 60 min of TGF-β1 treatment (Figures 2, 3). TGF-β1 recruited LMTK2 to the apical membrane by specifically stimulating Rab11-mediated endocytic trafficking of LMTK2 (Figure 5). By contrast, TGF-β1 did not inhibit LMTK2 endocytosis or LMTK2 trafficking in Rab5-positive endocytic vesicles (Figure 4), or induce LMTK2 protein degradation (Figure 2A). Finally, TGF-β1 did not increase LMTK2 mRNA level (Figure 2G).

The TGF-β family consists of several ligands playing an important role in lung physiology and pathophysiology (Bartram and Speer, 2004; Saito et al., 2018). The ligands are expressed at low levels in healthy adult lungs, playing a role in tissue repair after acute injury, while their expression increases in chronic lung disease (Magnan et al., 1994; Coker et al., 1996). The levels of different TGF-β ligands increase before development of abnormal lung function and correlate with the severity of chronic disease (Jagirdar et al., 1996; Minshall et al., 1997; Charpin et al., 1998; Salez et al., 1998). CF is a disease associated with increased levels of TGF-β1 due to *TGF-*β*1* gene polymorphisms, exposure to environmental toxins, including cigarette smoke, as well as infections with *Pseudomonas aeruginosa* or poor nutritional status. Prolonged exposure to TGF-β1 inhibits CFTR biogenesis by reducing mRNA stability and protein abundance in primary differentiated HBE cells from non-CF individuals and F508del homozygous patients (Snodgrass et al., 2013; Mitash et al., 2019) and in nasal polyps (Prulière-Escabasse et al., 2005), as well as to impair the functional rescue of F508del-CFTR (Snodgrass et al., 2013; Sun et al., 2014). However, acute effects of TGF-β1 on CFTR are unknown. Data presented in this manuscript close the gap showing that a short-term exposure to TGF-β1 may also inhibit CFTR mediated Cl^–^ transport. The specific mechanism, previously elucidated by our group, explains that LMTK2 phosphorylates CFTR-Ser^737^ at the apical membrane domain, leading to CFTR endocytosis and subsequent decrease of the CFTR-mediated Cl^–^ transport (Luz et al., 2014). Our data are clinically relevant to CF and other lung diseases. TGF-β1 impairs the corrector/potentiator-mediated rescue of F508del-CFTR *in vitro*, suggesting that it may prevent the full beneficial effect of the therapeutic approach in those CF patients who present with elevated TGF-β1 levels. Acquired CFTR dysfunction has been observed in COPD and the acute phase of pulmonary edema where TGF-β1 is a critical mediator (Clunes et al., 2012; Rab et al., 2013). Overall, our work highlights the relevance of assessing downstream mediators of TGF-β1-induced CFTR dysfunction in order to develop novel therapeutic approaches for different forms of lung diseases.

Small Rab-GTPases regulate endocytic trafficking in different vesicular compartments. Rab5 regulates trafficking from the plasma membrane to early endosomes (Bucci et al., 1992; Grant and Donaldson, 2009; Los et al., 2011), while Rab11 directs vesicles from the recycling endosomes to the plasma membrane, and also functions as an adaptor for endocytic trafficking from the *trans*-Golgi network to the plasma membrane (Ullrich et al., 1996; Grant and Donaldson, 2009; Los et al., 2011). We have shown that TGF-β1 modulates the endocytic trafficking pathway of LMTK2 in HBE cells. Specifically, TGF-β1 did not inhibit LMTK2 endocytosis nor did increase LMTK2 apical membrane abundance after silencing Rab5, indicating that it does not lead to apical membrane retention of LMTK2 by preventing its endocytosis. By contrast, silencing Rab11 blocked the TGF-β1 increase of apical membrane LMTK2, suggesting that TGF-β1 stimulates Rab11-mediated LMTK2 trafficking. We were unable to directly examine LMTK2 recycling using the GSH protection assay because of insufficient abundance of endogenous LMTK2 in the apical plasma membrane. Thus, we cannot distinguish whether the TGF-β1-mediated increase of apical membrane LMTK2 is caused by trafficking in recycling endosomes or by the vectorial transport from *trans*-Golgi network.

Whereas numerous studies investigated the activation and/or regulation of the TGF-β1 signaling pathway (Miyazono, 2000; Zhu and Burgess, 2001; Yan et al., 2018), as well as the regulation of the endocytic recycling trafficking of the TGF-β receptors (Mitchell et al., 2004; Chen, 2009; Penheiter et al., 2010; Yakymovych et al., 2018), currently, there are no published data demonstrating the role of TGF-β1 in trafficking of transmembrane proteins. Here, we addressed the research gap by unveiling that TGF-β1 regulates intracellular trafficking of LMTK2 leading to inhibitory phosphorylation and endocytosis of cell surface CFTR, and subsequent inhibition of CFTR-mediated Isc. This novel mechanism increases our understanding of the role of TGF-β1 in the pathophysiology of lung disease, including CF and the decreased efficacy of CFTR modulators in the presence of TGF-β1.

In summary, our data demonstrate that TGF-β1 recruits LMTK2 to the apical plasma membrane in HBE cells by increasing LMTK2 recycling in Rab11-positive vesicles. LMTK2 induces the inhibitory phosphorylation of CFTR-Ser^737^ triggering CFTR endocytosis and inhibition of CFTR-mediated Cl^–^ transport. Here, we propose a novel mechanism of TGF-β1-dependent inhibition of CFTR mediated by LMTK2 in human airway epithelial cells.

## Data Availability Statement

All datasets generated for this study are included in the article/supplementary material.

## Author Contributions

CF and AS-U designed the study, performed the experiments, analyzed the data, and wrote and reviewed the manuscript. DC performed the experiments, analyzed the data, and wrote and reviewed the manuscript. NM performed the experiments and analyzed the data. All authors read and approved the final version of the manuscript.

## Conflict of Interest

The authors declare that the research was conducted in the absence of any commercial or financial relationships that could be construed as a potential conflict of interest.

## Abbreviations

ALI: air-liquid interface
ASL: airway surface liquid
BSA: bovine serum albumin
cAMP: cyclic adenosine monophosphate
CF: cystic fibrosis
CFTR: cystic fibrosis transmembrane conductance regulator
EGFR: epidermal growth factor receptor
ENaC: epithelial sodium channel
ER: endoplasmic reticulum
ERK: extracellular signal-regulated kinase
FBS: fetal bovine serum
FDA: Food and Drug Administration
GSH: L-glutathione
HB: homogenization buffer
HBE: human bronchial epithelial cells
I_SC_: short circuit current
LMTK2: lemur tyrosine kinase 2
PBS: phosphate-buffered saline
PI3K: phosphatidylinositol 3-kinase
TGF: transforming growth factor
WCL: whole cell lysate
